# A Target Repurposing Approach Identifies N-myristoyltransferase as a New Candidate Drug Target in Filarial Nematodes

**DOI:** 10.1371/journal.pntd.0003145

**Published:** 2014-09-04

**Authors:** Brendan D. Galvin, Zhiru Li, Estelle Villemaine, Catherine B. Poole, Melissa S. Chapman, Michael P. Pollastri, Paul G. Wyatt, Clotilde K. S. Carlow

**Affiliations:** 1 New England Biolabs, Division of Genome Biology, Ipswich, Massachusetts, United States of America; 2 Northeastern University, Department of Chemistry and Chemical Biology, Boston, Massachusetts, United States of America; 3 Drug Discovery Unit, College of Life Sciences, University of Dundee, Dundee, Scotland, United Kingdom; University of Liverpool, United Kingdom

## Abstract

Myristoylation is a lipid modification involving the addition of a 14-carbon unsaturated fatty acid, myristic acid, to the N-terminal glycine of a subset of proteins, a modification that promotes their binding to cell membranes for varied biological functions. The process is catalyzed by myristoyl-CoA:protein N-myristoyltransferase (NMT), an enzyme which has been validated as a drug target in human cancers, and for infectious diseases caused by fungi, viruses and protozoan parasites. We purified *Caenorhabditis elegans* and *Brugia malayi* NMTs as active recombinant proteins and carried out kinetic analyses with their essential fatty acid donor, myristoyl-CoA and peptide substrates. Biochemical and structural analyses both revealed that the nematode enzymes are canonical NMTs, sharing a high degree of conservation with protozoan NMT enzymes. Inhibitory compounds that target NMT in protozoan species inhibited the nematode NMTs with IC_50_ values of 2.5–10 nM, and were active against *B. malayi* microfilariae and adult worms at 12.5 µM and 50 µM respectively, and *C. elegans* (25 µM) in culture. RNA interference and gene deletion in *C. elegans* further showed that NMT is essential for nematode viability. The effects observed are likely due to disruption of the function of several downstream target proteins. Potential substrates of NMT in *B. malayi* are predicted using bioinformatic analysis. Our genetic and chemical studies highlight the importance of myristoylation in the synthesis of functional proteins in nematodes and have shown for the first time that NMT is required for viability in parasitic nematodes. These results suggest that targeting NMT could be a valid approach for the development of chemotherapeutic agents against nematode diseases including filariasis.

## Author Summary

Lymphatic filariasis and onchocerciasis are neglected tropical diseases caused by filarial nematodes. The limitations of existing drugs to treat these infections highlight the need for new drugs. In the present study, we investigated myristoylation, a lipid modification of a subset of proteins that promotes their binding to cell membranes for varied biological functions. The process is catalyzed by N-myristoyltransferase (NMT), an enzyme which has been validated as a drug target in protozoan parasites. We performed kinetic analyses on *Caenorhabditis elegans* and *Brugia malayi* NMTs. NMT inhibitors were active against *B. malayi* microfilariae and adult worms, and *C. elegans* in culture. RNA interference and gene deletion in *C. elegans* further demonstrated that NMT is essential for nematode viability. Our genetic and chemical studies indicate the importance of myristoylation in the synthesis of functional proteins in nematodes and have shown for the first time that NMT is required for viability in parasitic nematodes. These results suggest that targeting NMT could be a valid approach for the development of new therapies against nematode infection including filarial diseases.

## Introduction

Nematode parasites are the causative agents of a large and diverse group of infectious diseases that affect millions of people, particularly in tropical and sub-tropical regions of the world. Lymphatic filariasis and onchocerciasis are chronic, disabling, neglected tropical diseases (NTDs) caused by filarial nematodes. Currently more than 1.4 billion people in 73 countries are threatened by lymphatic filariasis, with over 40 million incapacitated by the disease [1] Onchocerciasis occurs mainly in Africa with more than 99% of the 26 million infected people living in 31 countries in sub-Saharan Africa [2]. Mass drug administration (MDA) campaigns, involving annual large-scale treatment with albendazole together with either ivermectin (where onchocerciasis is endemic) or diethylcarbamazine citrate (where onchocerciasis is not present) to cover the entire at-risk population irrespective of disease status, form the foundation of attempts to control filarial infections. The drugs interrupt transmission by killing juvenile parasites but do not kill mature worms, and therefore multiple rounds of treatment are required before adult worms eventually die. In the absence of an adulticide, it is recommended that the MDA should be continued for 4–6 years for lymphatic filariasis [1] and 10–15 years for onchocerciasis [2]. Of particular concern to the MDA programs in Africa is co-endemic loiasis which can result in severe adverse neurological events following medication. The limitations of existing treatments and concerns for emergence of drug resistance [3] highlight the need for additional effective, safe and affordable drugs to treat the populations affected by filarial diseases.

One approach to anti-infective drug discovery involves target repurposing, where targets are selected based on their homology to a target for which a drug has already been identified for another species or indication. Existing knowledge of the biochemistry, structure and medicinal chemistry around the target is leveraged to enable rapid identification of new drug candidates. For example, the rapid development of HIV protease inhibitors was largely based on chemical expertise resulting from studies on human aspartic proteases [4], [5]. Target repurposing is a particularly attractive strategy for NTD drug discovery as it can provide an accelerated and more economical path to new treatments [6]. A key element to this approach is the identification of shared protein sequences among organisms, which has been greatly facilitated by the increased availability of genome sequences. Functional genomic studies in the pathogen or a model organism can validate candidate targets and guide a repurposing program.

The genome of the free-living nematode *Caenorhabditis elegans* has been used extensively as a model to understand molecular pathways common to nematodes and mammals on the basis of conserved evolution. For more than 30 years *C. elegans* has been employed in screening for anti-nematode compounds [7]. It has also been used in pharmacology studies [8] and to gain insight into the mechanism of action of known compounds, and the development of drug resistance [9]. The ability to perform forward and reverse genetic studies in *C. elegans* enables comprehensive validation of putative drug targets, which is key to the success of a target-based drug discovery approach. The abundance of functional genomic data from *C. elegans* and the high degree of genome sequence conservation between *C. elegans* and the human filarial parasite *Brugia malayi*, a causative agent of lymphatic filariasis, has enabled the prediction of potentially essential genes in *B. malayi* that may be exploited as drug targets [10].

The enzyme myristoyl-CoA:protein N-myristoyltransferase (NMT, EC 2.3.1.97) has been extensively investigated as a drug target in human cancer [11]–[13], and in infectious diseases caused by viruses [13]–[15], pathogenic fungi [16], and parasitic protozoa including *Trypanosoma brucei* [17]–[19], *Trypanosoma cruzi* [20], *Leishmania major* [18], *Leishmania donovani* [21], and *Plasmodium falciparum* [17], [18], [22]. NMT is an essential monomeric enzyme responsible for the co- and post-translational modification of proteins by transferring the fatty acid myristate (C_14.0_) from myristoyl-CoA to N-terminal glycine resides of a peptide/protein substrate, enabling their targeting to various membranes [23], [24] and/or activation and stabilization of the substrate protein [17]. Numerous biological studies have validated *T. brucei* NMT (TbNMT) as a druggable target using the prototypic TbNMT inhibitor DDD85646 [19], [23].

In this study we performed detailed molecular and biochemical studies on the NMT enzymes from *C. elegans* and the human filarial parasite *B. malayi*. We validated NMT as a new nematode drug target by performing genetic studies in *C. elegans*, and generated initial validation data using chemical inhibition studies with DDD85646 (and a related derivative DDD100870) in *C. elegans* and *B. malayi*. We also predicted the downstream target proteins in *B. malayi* that may be disrupted as a result of NMT inhibition. These studies represent the first analysis of N-myristoyltransferases from a helminth parasite, and demonstrate the importance of lipidation in nematodes including filarial worms. Our data strongly support repurposing N-myristoyltransferase for development of new therapies against nematode infection including filarial diseases.

## Materials and Methods

### Sequence and structural analysis of NMT proteins

Full-length NMT sequences from *Brugia malayi* (XP_001896037), *Caenorhabditis elegans* (NP_498326.1), *Loa loa* (XP_003141266.1), *Trypanosoma brucei* (EAN78792.1), *Plasmodium falciparum* (AAF18461.1), *Leishmania major* (AAG38102.1), *Saccharomyces cerevisiae* (NP_013296.1), *Homo sapiens* (AAH06376.1) and *Ascaris suum* (ERG81997.1) were retrieved from NCBI. *B. malayi* NMT was used to query additional databases to identify unannotated orthologs in other sequenced nematode genomes. The orthology assignments determined by TBLASTN analysis were recovered and contig sequences were assembled manually to produce a full-length protein sequence. The following list of nematode NMT proteins were manually curated in this manner:

*Wuchereria bancrofti* (http://www.broadinstitute.org/annotation/genome/filarial_worms/Blast.html?sp=Stblastn; contigs WUBG_03313.1, WUBG_18444.1, and WUBG_06452.1), *Trichinella spiralis* (https://www.wormbase.org/tools/blast_blat; GL622787, Length = 12041450), *Dirofilaria immitis* (http://xyala.cap.ed.ac.uk/downloads/959nematodegenomes/blast/filareu.php; nDi.2.2.scaf00055, Length = 228449), *Acanthocheilonema viteae* (http://xyala.cap.ed.ac.uk/downloads/959nematodegenomes/blast/filareu.php; nAv.1.0.scaf00057, length = 84838), *Litomosoides sigmodontis* (http://xyala.cap.ed.ac.uk/downloads/959nematodegenomes/blast/filareu.php; nLs.2.1.scaf00231, length = 65134 and nLs.2.1.scaf00078, length = 103883), *Onchocerca volvulus* (http://xyala.cap.ed.ac.uk/downloads/959nematodegenomes/blast/filareu.php; nOv_contig22006, length = 20337), and *Onchocerca ochengi* (http://xyala.cap.ed.ac.uk/downloads/959nematodegenomes/blast/filareu.php; nOo.2.0.Scaf00398, length = 32289).

Protein alignment was performed using the ClustalW alignment software (http://www.genome.jp/tools/clustalw/) and displayed using BOXSHADE (http://www.ch.embnet.org/software/BOX_form.html). The residues involved in binding of *L. major* NMT to myristoyl-CoA and DDD85646 determined from co-crystal structural analyses were obtained from: http://www.rcsb.org/pdb/explore/explore.do?structureId=2wsa. A rooted phylogenetic tree with branch length was generated using the UPGMA (**U**nweighted **P**air **G**roup **M**ethod with **A**rithmetic Mean) ClustalW software. Sequence identity values between *B. malayi* and an ortholog were generated using BLASTP.

A homology model of the *B. malayi* NMT structure was built based on several structurally characterized NMT's, including enzymes from *Leishmania donovani* (2wuu), *Plasmodium vivax* (4a95), and *Saccharomyces cerevisiae* (2p6f) using the PHYRE program (http://www.sbg.bio.ic.ac.uk/phyre2/html/page.cgi?id=index). The FASTA sequence for *B. malayi* was uploaded to the Protein Homology/analogY Recognition Engine V 2.0 (Phyre2) server, where models were automatically generated using the best possible template model [25]. The predicted structure of *B. malayi* NMT was then compared to the structure of *L. major* NMT (2wsa) bound to myristoyl-CoA and inhibitor DDD85646 using the UCSF Chimera software [26].

### Expression and purification of nematode NMT enzymes

Full-length *CeNMT* cDNA was reverse transcribed from total *C. elegans* RNA and then amplified using the following primers: (GATCGGGAATTCATATGTCCCACGGACACAGTC) and (GATCCCGCTCGAGTTGAAGAACAAGCCCGATTT) containing a NdeI or XhoI restriction site (underlined), respectively, to enable cloning into the corresponding sites of the expression vector. *BmNMT (Bm1_22900)* was a synthetic (codon-optimized) version of the gene (GenScript Corporation, Piscataway, NJ) designed to optimize expression in *E. coli*. Each nematode NMT gene was cloned into the pET19b vector to express a fusion protein with a 10-His tag at the N-terminus. The insert was then fully sequenced for verification. Plasmids were transformed into the *E. coli* strain NiCo21(DE3) (NEB) for protein expression. Cultures were grown at 37°C and induced with 0.4 mM IPTG at 16°C overnight. The His-tagged proteins were extracted in lysis buffer (20 mM NaH_2_PO_4_, 300 mM NaCl, 10 mM imidazole, pH 8.0) containing protease inhibitors (Complete EDTA-free Protease Inhibitor, Roche), and purified on 1 mL HisTrap HP (GE Healthcare) using an ÄKTA-FPLC system (GE Healthcare). Protein was eluted using a linear (0–100%) gradient of elution buffer (20 mM NaH_2_PO_4_, 300 mM NaCl, 250 mM imidazole, pH 8.0). Fractions containing NMT were pooled and dialyzed into storage buffer (20 mM Tris-HCl, 100 mM NaCl, 2 mM DTT, 2 mM EGTA, 2 mM EDTA, and 50% glycerol, pH 7.5).

### Biochemical assays

Nematode NMT proteins were assayed using synthetic peptides GGVMSYRRR (ARL-1 ADP ribosylation factor related protein), GHSHSTGKRR (ABL-1 tyrosine kinase) and GCLFSKERR (SRC-1 tyrosine kinase), derived from N-terminal sequence of several *C. elegans* N-myristoylated proteins. One or two basic amino acids (underlined) were added at the C-terminal end of the peptide to generate a positive charge at pH 7.3. The NMT assay was performed as described with some minor modification [18], [27]. Reactions were carried out in a final volume of 50 µL containing 30 mM Tris-HCl buffer (pH 7.5), 0.5 mM EDTA, 0.45 mM EGTA, 4.5 mM 2-mercaptoethanol, 1% Triton X-100, 1 mM peptide and 5 ng purified enzyme. N-myristoylation was initiated by the addition of 0.5 mM myristoyl-CoA. Myristoyl-CoA was prepared by mixing equal volumes of cold myristoyl-CoA (1 mM, Chem-Impex International) with [^3^H] myristoyl-CoA (5 µM, 42 Ci/mmol, Perkin Elmer). After a 10 minute incubation at 30°C, the reaction was terminated by spotting 25 µL reaction mixture onto P81 phosphocellulose paper (Whatman). The paper was dried under a heat lamp and washed three times for 10 minutes in 20 mM Tris-HCl pH 7.5 prior to scintillation counting using a Liquid scintillation Analyzer (Tri-Carb 2900 TR, Perkin Elmer). All reactions were performed in duplicate, and reactions performed in the absence of enzyme, or peptide, were included as controls. Background radioactivity values generated from control samples were subtracted from the values obtained from experimental samples.

To determine the kinetic constants: K_m_, V_max_, K_cat_ and K_cat_/K_m_ of *B. malayi* and *C. elegans* NMT for myristoyl-CoA, the concentration of myristoyl Co-A was varied from 0.1–50 µM in the presence of 1 mM peptide (ABL-1) and 5 ng enzyme. Reactions were performed in triplicate. Optimum fitting of data to the Michaelis-Menten equation was calculated using non-linear regression software (http://www.colby.edu/chemistry/PChem/scripts/lsfitpl.html). One unit of NMT activity is defined as 1 pmol of myristoylated peptide formed per min.

Inhibition assays for compounds DDD85646 and DDD100870 were performed in a final volume of 50 µL containing 10 mM Tris pH 7.5, 0.5 mM DTT, 0.1 mM EDTA, 10 nM enzyme and 5 µM myristoyl-CoA (as above). Enzyme mixtures were preincubated with various concentrations of each compound (0.0025–1 µM) dissolved in DMSO for 7.5 min at 25°C then the reactions were initiated by adding peptide substrate (ABL-1, 12.5 µM). Following a 15 minute incubation at 25°C, reactions were terminated by spotting 25 µL onto P81 phosphocellulose paper, washed, dried and prepared for scintillation counting as described previously. Reactions performed in the absence of inhibitor or peptide were included as controls. Experiments were performed in triplicate. Mean IC_50_ values were determined in the concentration range of 2.5–10 nM.

### *C. elegans* methods

*C. elegans* culture and handling were performed following standard procedures [28]. N2 strain var. Bristol was used as wild-type [29]. Mutant strains (NL4256 *rrf-3(pk1426*), *lin-15b(n744);eri-1(mg366)*) were obtained from the *C. elegans* stock center (CGC, University of Minnesota) and also (*nmt-1(tm796)/hT2[qla48]*) from Dr. S. Mitani (National Biosource Project of Japan, Tokyo Women's Medical University School of Medicine, Tokyo).

### Establishing the essential function of NMT in *C. elegans*

Three *C. elegans* strains were used for RNAi knockdown of NMT: *C. elegans* wild-type and two RNAi sensitive *C. elegans* strains, one containing a mutation in *rrf-3* [30], and a second strain carrying mutations in both *eri-1* and *lin-15B I* [31]. RNAi was performed by feeding an *Escherichia coli* strain expressing dsRNA [32]. Full-length NMT cDNA was subcloned into the feeding vector pL4440. HT115 *E. coli* transformed with this construct were spread on agar plates containing NGM supplemented with 1 mM isopropylthio-β-D-galactoside (IPTG, Sigma) and 50 µg/mL ampicillin, and incubated overnight at room temperature to induce dsRNA expression. RNAi assays were carried out by feeding L4-staged worms HT115 *E. coli* expressing dsRNA corresponding to NMT or pL4440 plasmid vector without *CeNMT*. The relative health of the progeny of each worm was determined qualitatively by its appearance relative to the controls over the course of several days. For each RNAi experiment, 50 worms were assayed from each of the 3 strains.

Phenotypic analyses were also performed using the mutant strain *nmt-1(tm796)/hT2[qls48]*. To verify the deletion, genomic DNA was prepared from a single mutant animal, and one worm from heterozygous and wild-type strains using standard methods [33]. The deletion was confirmed on the basis of the change in size of a PCR product using forward primer TCAACTGATTGCACCGTCAT and reverse primer AAGCGGAACATGGAATCATC. PCR reactions were carried out using 25 picomoles of each primer and LongAmp DNA Polymerase (NEB). Bands of the expected sizes were obtained.

### Testing compounds for activity against *C. elegans* and *B. malayi*

Wild-type *C. elegans* were grown on NGM plates seeded with OP50 *E. coli*. Compound screening commenced with L4-staged worms placed into a well of a sterile 96-well micro titer plate (Falcon 3072) containing a 100 µL suspension of previously frozen HB101 *E. coli* bacteria in S medium. Various concentrations of compound (25, 50 or 100 µM) or DMSO (control) were then added. Plates were maintained at 20°C in a humidity chamber for 7 days. Worm growth and development was scored daily by measuring a decrease in OD_600 nm_ resulting from consumption of *E. coli*, and by microscopic examination of the size and number of F1 progeny produced on day 3 of the experiment. For each condition, 10 L4-stage worms were used, and the average ± standard deviation was plotted.

Living *B. malayi* worms including adult female, adult male and microfilariae were purchased from TRS labs (Athens, GA). Worms were washed extensively with RPMI 1640 medium prior to culture in RPMI 1640 medium supplemented with 2 mM glutamine, 10% Fetal Calf Serum (Gibco) and 100 µg/mL streptomycin, 100 units/mL penicillin, 0.25 µg/mL amphotericin B (Sigma) at 37°C, in 5% CO_2_. After overnight recovery, adult worms were separated into 3 different groups each containing either 8 females (2 worms/well) or 12 males (3 worms/well). Compounds (DDD85646 or DDD100870) at 100 µM or 50 µM, or 1% DMSO were added to the culture medium. Microfilariae were cultured in 24-well plates and compounds were added to the culture medium at a final concentration of 100 µM, 50 µM, 25 µM or 12.5 µM. Experiments were performed in at least triplicate. The culture media were replaced every other day with fresh media containing compound or DMSO only. Parasite motility was video-recorded daily and analyzed at the end of the experiment (day 7). Parasite motility was assessed and scored in the range 0–20 (most motile). Observations are expressed as a percentage of the motility relative to the motility scored on day 0 of the experiment. Counting numbers of parasites on days 1, 3 and 5 assessed production of microfilariae from female worms. The data obtained from triplicate samples are expressed as mean ± standard deviation.

### Bioinformatic prediction of *B. malayi* NMT substrates

To identify potential targets for N-terminal glycine myristoylation in *B. malayi*, predicted myristoylated proteins in the proteome of *C. elegans* were retrieved (http://mendel.imp.ac.at/myristate/myrbase/28731NEgenpept227MYRclusterV05.html) and used to query the genome of *B. malayi*. Homologs with a BLASTP E-Value <e^−10^ were analyzed using the MYR predictor (http://mendel.imp.ac.at/myristate/SUPLpredictor.htm) to predict myristoylation sites. Protein sequences that were scored as “Reliable” or “Twilight zone” were retained and duplicates were discarded.

## Results

### Distribution and conservation of N-myristoyltransferases in nematodes

**Figure 1:**
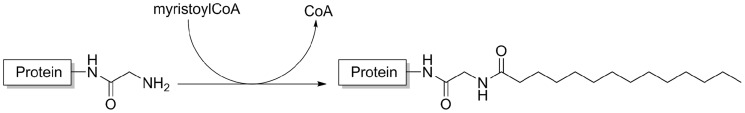
Reaction catalyzed by Myristoyl CoA:protein N-myristoyltransferase (NMT). NMT catalyzes the covalent attachment of fatty acid myristate to the N-terminal glycine of a subset of proteins. The fatty acid moiety is provided by myristoyl-CoA, which binds to the apo-enzyme first, forming a binary complex. This complex then binds the substrate protein and the reaction occurs. Subsequently, CoA is released, followed by release of the myristoylated protein product [17].

**Figure 2:**
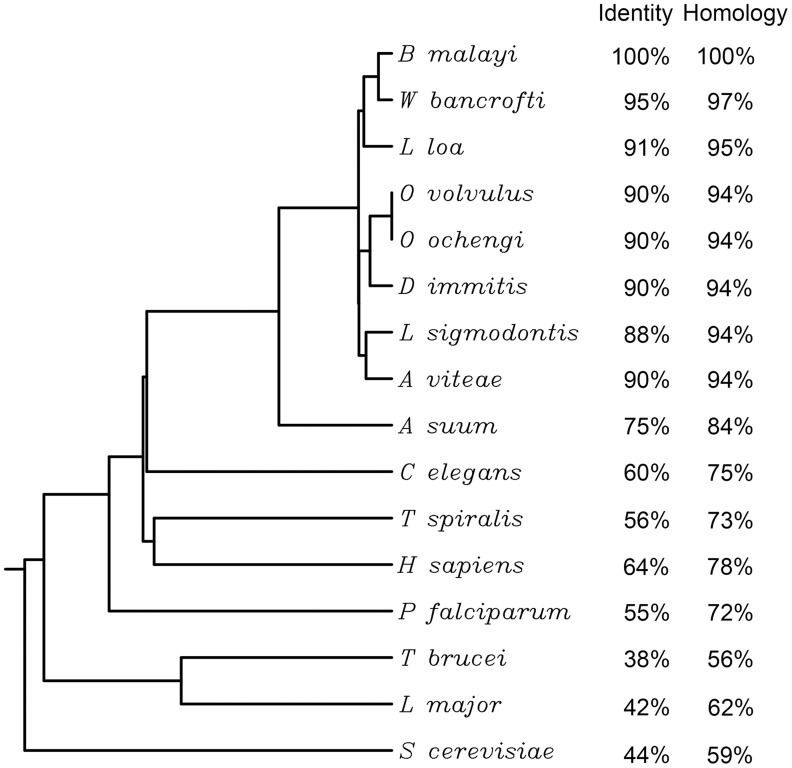
Evidence for the presence of N-myristoyltransferase (NMT) in nematodes and conservation of NMT among prokaryotes and eukaryotes. Rooted phylogenetic tree analysis of NMT and various homologs. Deduced open reading frames of NMTs were used to generate the phylogram. Branch length was generated using ClustalW (http://www.genome.jp/tools/clustalw/). The percentage of amino acid identity and similarity between *B. malayi* NMT and orthologs are shown.

Both *C. elegans* and *B. malayi* genomes contain a single copy *NMT* gene predicted to encode a N-myristoyltransferase which catalyzes the covalent attachment of fatty acid myristate to the N-terminal glycine of proteins (**Fig. 1**). In *C. elegans*, gene *T17E9.2* encodes three isoforms: A (450 aa, 51 kDa, pI 7.6), B (452 aa, 51 kDa, pI 7.9) and C (403 aa, 46 kDa, pI 8.6). All isoforms share the same amino acid sequence, with isoforms A and B possessing an additional 47 or 49 amino acids at the N-terminus, respectively. *BmNMT* encodes one 472 aa protein (XP_001896037) of 54 kDa with a pI of 6.6. *C. elegans* and *B. malayi* NMTs share approximately 60% amino acid identity and 75% similarity to each other (**Fig. 2**).

To investigate if NMT is present in other nematodes, *BmNMT* was used as a query to search ∼300,000 EST sequences at Nematode.net [34] that are available for 30 different parasitic nematodes species. Matching NMT sequences were found in free-living nematodes and a highly diverse group of parasitic nematode species found in humans (*Strongyloides stercoralis*), animals (*Strongyloides ratti*, *Ancylostoma caninum*, *Teladorsagia circumcincta*) and plants (*Meloidogyne hapla, Heterodera glycines*) representing four major clades of the phylum Nematoda (data not shown). To determine the degree of conservation of the enzyme among nematodes, full-length sequences (**Fig. S1**) were obtained for a number of species (*B. malayi*, *C. elegans*, *Loa loa*, *Ascaris suum*, *Wuchereria bancrofti*, *Trichinella spiralis*, *Dirofilaria immitis*, *Acanthocheilonema viteae*, *Litomosoides sigmodontis*, *Onchocerca volvulus* and *Onchocerca ochengi*). The relationship of the nematode enzymes to other eukaryotic (*Homo sapiens*, *Saccharomyces cerevisiae*) and prokaryotic (*Trypanosoma brucei*, *Plasmodium falciparum*, *Leishmania major*) NMTs was also examined. Phylogenetic tree analyses (**Fig. 2**) indicated that the filarial nematode sequences (Clade III) form a close cluster with a high degree of conservation (greater than 88% identity, 94% similarity). *Trichinella spiralis* (Clade I) and *Ascaris suum* (Clade III) enzymes are 73% and 84% similar to *B. malayi* NMT, respectively. *B. malayi* NMT also shares significant similarity to protozoan (56–62%), human (64%) and yeast (59%) enzymes.

Multiple sequence alignment of *B. malayi* NMT and orthologs from *Loa loa*, *O. volvulus*, *C. elegans*, *S. cerevisiae*, *L. major*, *T. brucei* and *H. sapiens* also revealed conservation in the residues involved in binding to myristoyl-CoA (**Fig. S2**) [23]. Consistent with other NMT enzymes, the nematode NMTs are divergent at their N-termini, a region not involved in substrate binding [21], [23], and all possess an N-terminal extension not present in the previously studied NMTs from protozoa or yeast.

**Figure 3:**
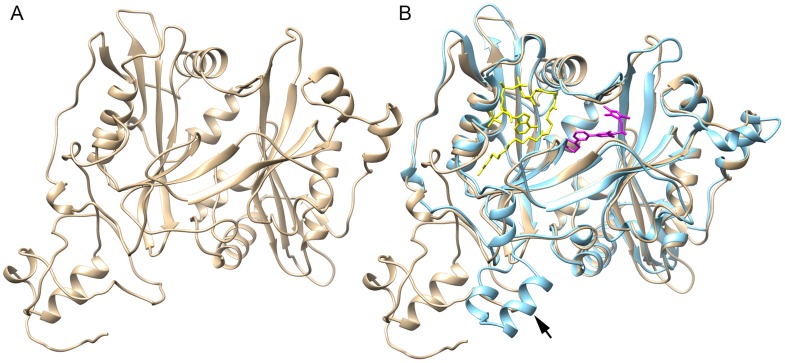
Modeling the structure of *B. malayi* NMT and structural comparison with *Leishmania major* NMT. (A) Ribbon diagram of the predicted crystal structure of NMT from *B. malayi*. (B) Comparison of *B. malayi* NMT (tan) with *L. major* NMT (blue: 2wsa). An overlay of the structure of these enzymes using UCSF Chimera [26] reveals a nearly identical conformation of the binding sites for myristoyl-CoA (yellow) and inhibitor DDD85646 (magenta). The 2 small helixes (arrow) formed by an insertion of 21 amino acids in *L. major* NMT are replaced with a loop in *B. malayi* NMT.

A homology model indicates that *B. malayi* NMT possesses a canonical NMT structure with a typical NMT fold characteristic of the Acyl-CoA N-acyltransferases superfamily [35]. The NMT fold basically consists of a large saddle-shaped β-sheet that is flanked on both of its faces by several helices (**Fig. 3A**). The model generated is based on the structurally characterized NMTs from *Saccharomyces cerevisiae* (c2p6fA) and *Leishmania donovani* (2wuu), and is predicted to have a 100% confidence level.

When the predicted *B. malayi* NMT structure is compared with the structure of the *Leishmania major* NMT (2wsa) enzyme bound to myristoyl-CoA and inhibitor DDD85646, a high degree of conservation was revealed in the myristoyl-CoA binding site as well the drug binding pocket (**Fig. 3B**). The 2 small helixes (**Fig. 3B** **, arrow**) formed by an insertion of 21 amino acids in *L. major* NMT are replaced with a loop in *B. malayi* NMT that is unlikely to have any major effects on the binding of substrate or inhibitor.

### Recombinant nematode NMT proteins are active *N*-myristoyltransferases

**Figure 4:**
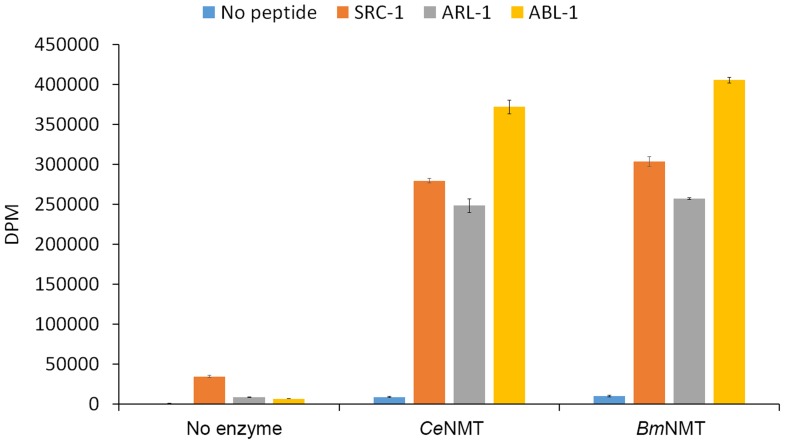
Biochemical analysis of recombinant *C. elegans* NMT and *B. malayi* NMT. Purified recombinant *B. malayi* NMT and *C. elegans* NMT enzymes myristoylate several synthetic peptide substrates (ARL-1 ADP ribosylation factor related protein; ABL-1 tyrosine kinase and SRC-1 tyrosine kinase). No activity was detected in the absence of peptide. Enzyme activity is expressed as radioactivity in disintegrations per minute (DPM).

To verify their NMT activities, recombinant *C. elegans* NMT (51 kDA) and *B. malayi* NMT (54 kDA) were expressed, purified and assayed using several synthetic peptides containing a canonical myristoylation motif derived from the N-terminal sequence of three *C. elegans* proteins. *BmNMT (Bm1_22900)* was a synthetic (codon-optimized) version (**Fig. S3**) of the gene (GenScript Corporation, Piscataway, NJ) designed to optimize expression in *E. coli*. Expression studies using *BmNMT* cDNA isolated by RT-PCR (**Fig. S3**) generated largely insoluble protein. Both recombinant proteins displayed similar levels of activity using the various peptides, with the highest activity observed with ABL-1 (**Fig. 4**).

Kinetic analyses of recombinant nematode NMTs were performed using ABL-1 as the peptide. The K_m_, V_max_, K_cat_ and K_cat_/K_m_ for myristoyl-CoA were determined for both enzymes (**Fig. S4**). The *C. elegans* K_m_ = 12.2±0.91 µM) and *B. malayi* (K_m_ = 4.4±0.12 µM) enzymes showed similar affinities for myristoyl-CoA. K_cat_/K_m_ values were also comparable for both *C. elegans* (0.13 s^−1^ µM^−1^) and *B. malayi* (0.15 s^−1^ µM^−1^) NMTs. While these values cannot be compared directly with those measured for the NMTs of other species as the assay substrates and conditions are different, the data obtained are of the expected order of magnitude.

### Inhibition of nematode NMT activity using anti-trypanosome compounds

**Figure 5:**
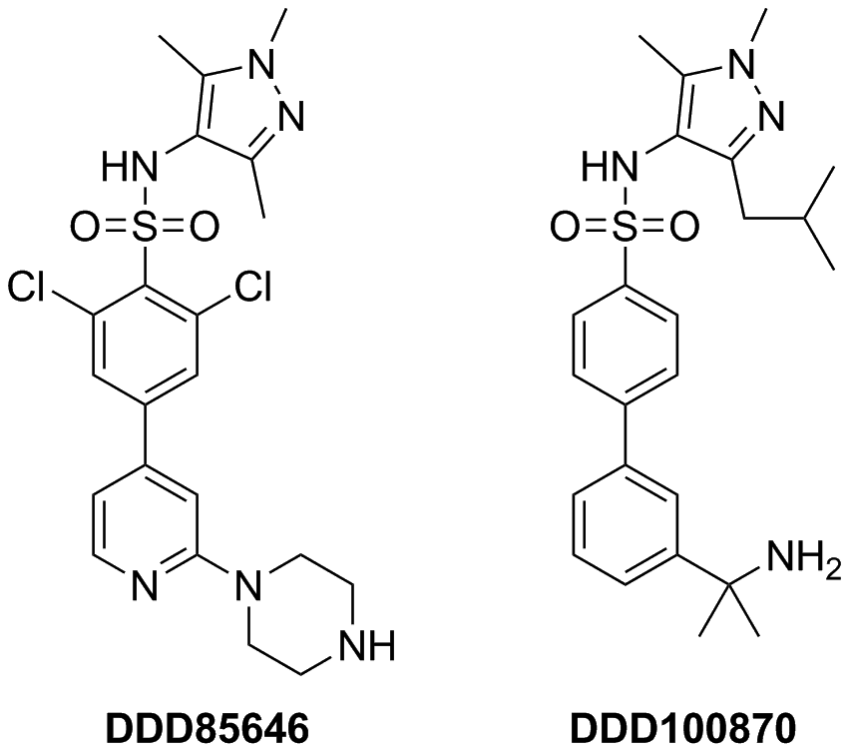
Structures of the NMT inhibitors, DDD85646 and DDD100870.

**Figure 6:**
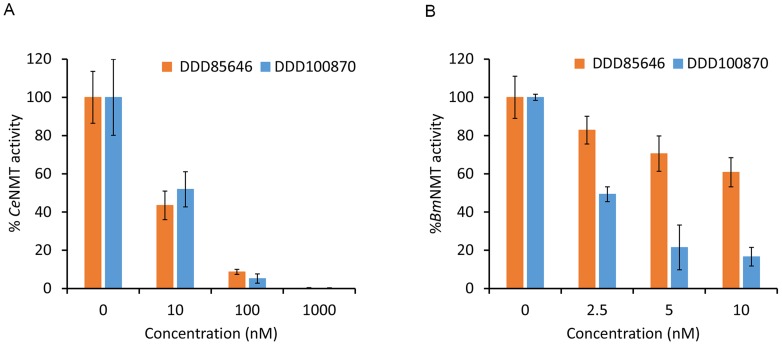
Inhibition of *C. elegans* (A) and *B. malayi* (B) NMTs using DDD85646 and DDD100870. Enzyme mixtures were preincubated with various concentrations of each compound (0.0025–1 µM). Reactions performed in the absence of inhibitor (positive control) or peptide (negative control), were included. Background radioactivity values generated from ‘non-peptide’ control samples were subtracted from the values obtained from experimental samples. Percent activity relative to the positive control ± propagation of error (http://laffers.net/blog/2010/11/15/error-propagation-calculator/) was determined. Assays were performed in triplicate.

DDD85646 is a known inhibitor of NMT enzymes from several trypanosomatids. DDD85646 inhibits the proliferation of *T. brucei* in culture with >200 fold selectivity over mammalian cells [19], [23]. Given the degree of conservation observed in comparative sequence and structural analyses between nematode and trypanosome NMT enzymes, we examined the potency of DDD85646 and its analogue DDD100870 (**Fig. 5**) against *C. elegans* and *B. malayi* NMT proteins (**Fig. 6**). A dose-dependent inhibition of *C. elegans* NMT activity with an IC_50_ value of ∼10 nM was observed for both compounds (**Fig. 6A**). The IC_50_ value of DDD85646 for *B. malayi* NMT is also ∼10 nM. DDD100870 was a more potent inhibitor of the filarial enzyme since an IC_50_ value of 2.5 nM was obtained.

### *C. elegans* NMT plays an essential role in development

**Figure 7:**
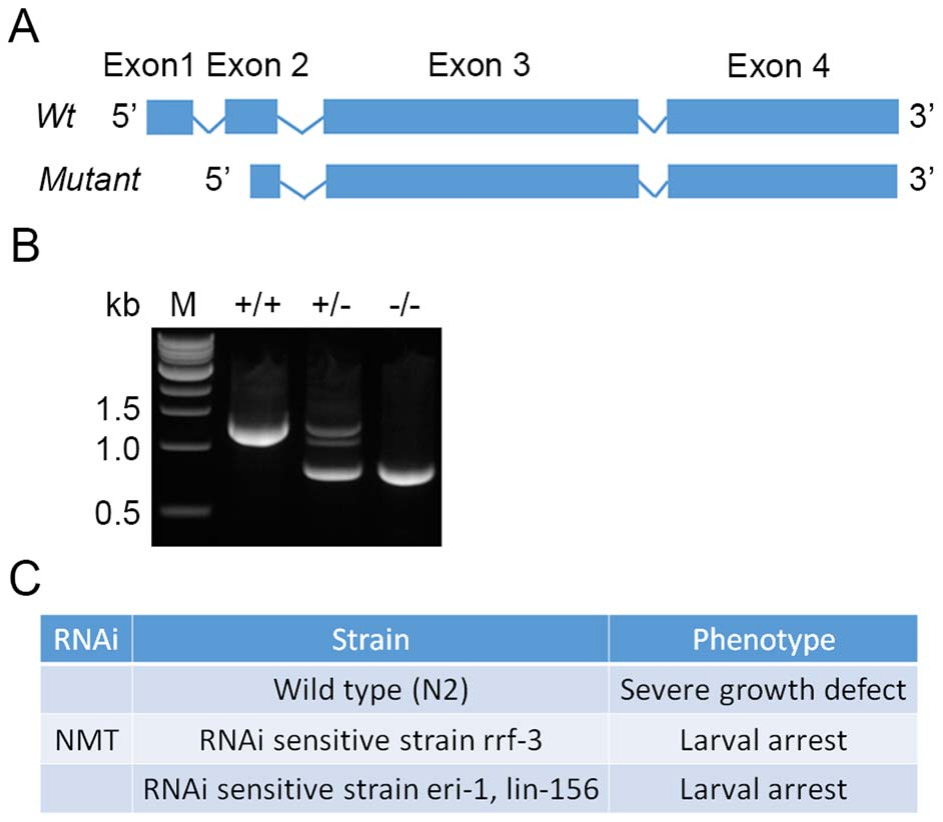
NMT is essential in *C. elegans*. (A) Phenotypic analyses were performed using the mutant strain *nmt-1(tm796)/hT2[qla48]*. Predicted genomic organization of *CeNMT* and location of the deletion are shown. Boxes and lines denote exons and introns respectively. To verify the deletion genomic DNA was prepared from a single mutant animal “−/−”, and one worm from heterozygous “+/−” and wild-type “+/+” strains using standard methods. (B) Primers were designed to flank the deletion site and the deletion was confirmed on the basis of the change in size of a PCR product. Bands of the expected sizes were obtained. (C) RNAi knockdown of NMT in *C. elegans*. Three *C. elegans* strains were used for RNAi knockdown of NMT: *C. elegans* wild-type and two RNAi sensitive *C. elegans* strains, one containing a mutation in *rrf-3*, and a second strain carrying mutations in both *eri-1* and *lin-15B*. RNAi was performed by feeding worms *E. coli* expressing dsRNA corresponding to NMT, or pL4440 plasmid vector without *CeNMT*.

To investigate the importance of *C. elegans* NMT *in vivo*, phenotypic analyses were performed on a strain carrying a deletion allele (*nmt-1(tm796)/hT2[qls48]*) (**Fig. 7A, B**) and on worms with reduced endogenous activity resulting from RNAi [36]. The deletion was confirmed by PCR analysis. DNA prepared from wild-type and heterozygous worms generated a band of approximately 1295 bp, whereas a smaller band (799 bp) was obtained from both heterozygous and mutant animals (**Fig. 7B**). The *C. elegans nmt* gene comprises four exons and the size of the smaller band is consistent with a deletion involving exon 1 and exon 2 (partial) (**Fig. 7A**). Three *C. elegans* strains were used for RNAi knockdown of NMT: *C. elegans* wild-type and two RNAi sensitive *C. elegans* strains, one containing a mutation in *rrf-3* [30], and a second strain carrying mutations in both *eri-1* and *lin-15B* [31]. Homozygous *nmt-1(tm796)/hT2[qla48]* animals had an obvious phenotype marked by a maternal effect larval lethality with no viable worms present in the F2 population. In wild-type *C. elegans*, RNAi knockdown of *CeNMT* expression resulted in a severe growth defect, while in the RNAi sensitive strains (*rrf-3* and *eri-1*; *lin-15B*) larval arrest was observed (**Fig. 7C**). No abnormal phenotype was observed for these three strains when fed *E. coli* containing the pL4440 plasmid without *CeNMT*. These data demonstrate depletion of NMT activity in nematodes causes severe developmental defects and establishes the requirement of NMT for viability in *C. elegans*.

### Inhibition of nematode growth using NMT inhibitors

**Figure 8:**
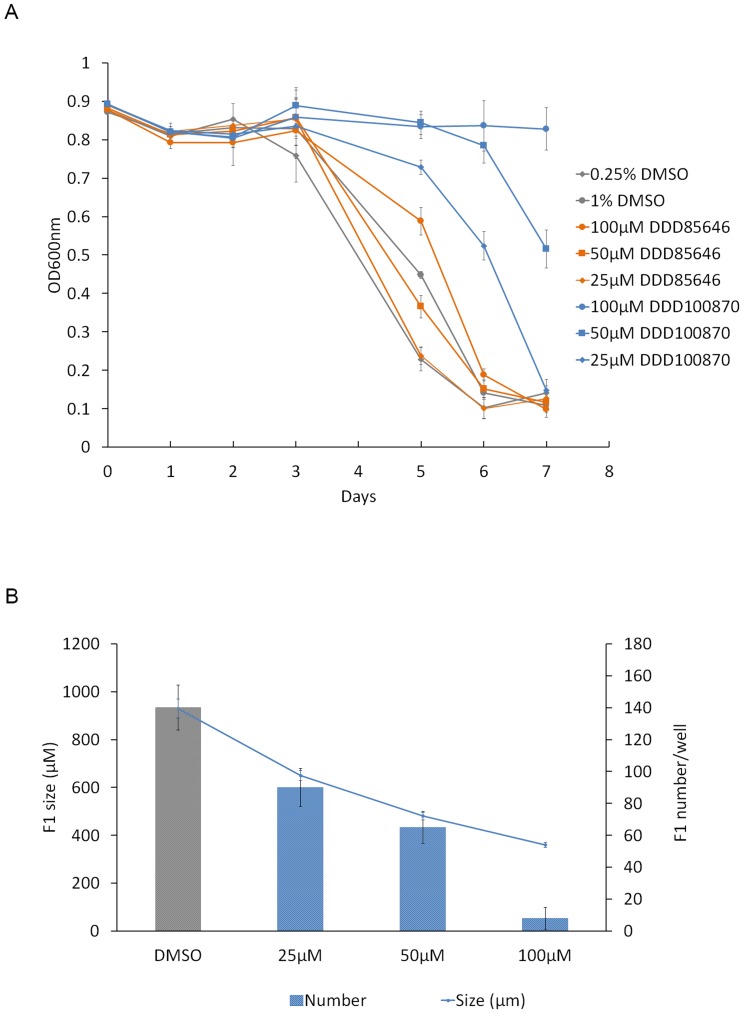
Effect of NMT inhibitors on *C. elegans* growth. Wild-type *C. elegans* were grown on NGM plates seeded with OP50 *E. coli*. Compound screening (DDD85646 and DDD100870) commenced with L4-staged worms placed into a well of a sterile 96-well micro titer plate (Falcon 3072) containing a 100 µL suspension of previously frozen HB101 *E. coli* bacteria in S medium. Various concentrations (25, 50 or 100 µM) of compound or DMSO (control) were then added. Plates were maintained at 20°C in a humidity chamber for 7 days. Worm growth and development was scored daily by measuring a decrease in OD_600 nm_ resulting from consumption of *E. coli*, (A) and by microscopic examination of the number of F1 progeny produced and size of worms treated with DDD100870 (B). For each condition, 10 L4-stage worms were used, and the average ± standard deviation was plotted.

The potent activities of DDD85646 and DDD100870 in nematode NMT activity assays and requirement of NMT for viability in *C. elegans* prompted evaluation of the compounds for *in vivo* activity against *C. elegans* and *B. malayi*. *C. elegans* L4s were treated with 25, 50 or 100 µM of each compound and worm growth and development was scored daily by measuring a decrease in OD_600 nm_ resulting from consumption of *E. coli* (**Fig. 8A**) and by microscopic examination of the size and number of F1 progeny (**Fig. 8B**). In control worms exposed to DMSO alone there was a rapid consumption of the *E. coli* food source as indicated by a decline in OD_600_ value. DDD100870 treated worms showed slower feeding/growth that was dose dependent, with 100 µM compound resulting in almost complete inhibition of feeding. In contrast, 100 µM of the related compound DDD85646 only slightly delayed the clearance of bacteria from the culture. On day 3, DDD100870 treated worms showed a concentration dependent decline in the number of F1 progeny produced (**Fig. 8B**) while no such effect was observed for DDD85646 (data not shown). The progeny of controls averaged (140 worms) 930 µm in length, whereas worms treated with 100 µM DDD100870 resulted in no progeny or fewer progeny of smaller size (360 µm long). Worms exposed to 50 and 25 µM DDD100870 showed a dose-dependent decline in the number of F1 progeny produced and their size.

**Figure 9:**
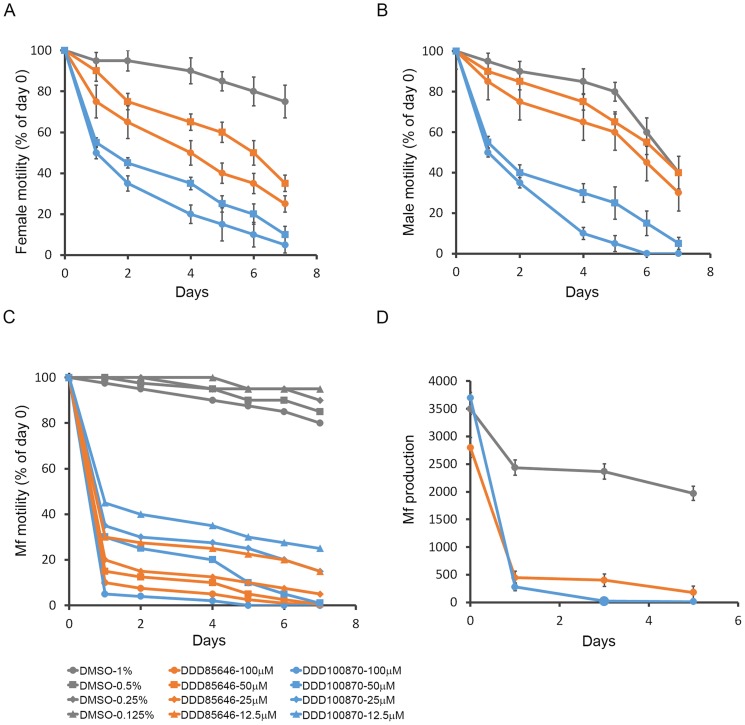
Effect of NMT inhibitors on *B. malayi in vitro*. Living *B. malayi* worms including adult female (A), adult male (B) and microfilariae (C) were exposed to NMT inhibitors for 7 days. Compounds DDD85646 (orange) or DDD100870 (blue) at 100 µM (circle) or 50 µM (square) or 1% DMSO (grey) were added to adult worm culture. Microfilariae (mf) were cultured in the presence of compounds at a final concentration of 100 µM (circle), 50 µM (square), 25 µM (diamond) or 12.5 µM (triangle). Experiments were performed in triplicate. The culture media were replaced each day with fresh media containing compound or DMSO (grey) at corresponding concentrations. Parasite motility was video-recorded daily and observations expressed as a percentage of the motility relative to the motility scored on day 0 of the experiment. Production of microfilaria from female worms (D) was assessed on days 1, 3 and 5. The data obtained from triplicate samples are expressed as mean ± standard deviation.

Adult worms and microfilariae of *B. malayi* were cultured in the presence of both NMT inhibitors (**Fig. 9**). The parasites were observed for changes in motility and production of microfilariae by adult female worms compared to control worms exposed to 1% DMSO. Both DDD85646 and DDD100870 exerted a significant and profound effect on *B. malayi*, though DDD100870 (effective against *C. elegans*) was more potent than DDD85646 (**Fig. 9**). A decline in the motility of exposed adult female (**Fig. 9A**) and male (**Fig. 9B**) worms was apparent within 24 hours of treatment. By day 6, DDD100870-treated adult worms showed little movement, while the control worms still displayed vigorous activity. Interestingly, DDD85646 displayed more potent activity against female worms than male worms. Microfilariae were more sensitive than adult worms to both compounds with concentrations as low as 12.5 µM immobilizing the worms within 24 hours of exposure (**Fig. 9C**). Microfilariae production by female worms was also severely affected by treatment and a decline was apparent at 24 hours post culture (**Fig. 9D**).

### Potential N-myristoylation substrates in *B. malayi*

To predict downstream target proteins that may be disrupted due to NMT inhibition by compounds DDD85646 and DDD100870, a bioinformatics approach was used to identify potential targets for N- myristoylation in *B. malayi*. Predicted myristoylated proteins in the proteome of *C. elegans* (145 clusters) were retrieved from MYRbase and used to query the *B. malayi* genome. A total of 40 unique protein sequences were obtained; 30 scored “reliable” and 10 designated “twilight zone” (**Table 1**). The putative substrates are involved in diverse and essential pathways. These include ADP-ribosylation factors, hydrolases, protease activity, receptor activity and several are protein kinase domain containing proteins. In *C. elegans*, the majority of the predicted substrates (31/40) display multiple developmental defects and impaired survival in RNA interference assays (**Table 1**), and include the phenotypes we observed in RNAi knockdown of *CeNMT* and in mutant worms carrying a deletion allele. We cannot exclude the possibility that NMT has unknown functions in addition to myristoyltransferase activity. However, it is most likely that the pleiotropic effects observed are likely caused by inhibition of myristoylation of multiple important substrates, demonstrating the requirement of protein myristoylation in nematodes.

**Table 1:** Listing of predicted *B. malayi* NMT substrates.

*B. malayi* locus	Myristoylation motif	Score	Protein information	*C. elegans* homolog
Bm1_33335	GGNNSTPKSDTMMDHQP	3.982	hypothetical protein	F46C8.3
Bm1_01010	GCTMSQEERAALERSRM	3.961	guanine nucleotide-binding protein G-o protein alpha subunit	C26C6.2 *
Bm1_20595	GNYKSRPPSSCADELKK	3.617	Protein kinase domain containing protein	C24A1.3a *
Bm1_07670	GGRQSSQVSHEVAQSEV	3.327	cytochrome C-type heme lyase	T06D8.6 *
Bm1_36040	GNCFSGSPRQSSPSSDI	2.626	SRC-1, putative	Y92H12A.1 *
Bm1_54125	GNSGSTNIPGGGTEGYH	2.584	golgi reassembly stacking protein 2, putative	Y42H9AR.1
Bm1_51340	GSCQSQEAQEQLARNKA	2.509	Guanine nucleotide-binding protein alpha-3 subunit, putative	C34D1.3 *
Bm1_52520	GQQQSGFGGKREDRGGG	2.411	Probable 26S protease regulatory subunit 4	F29G9.5 *
Bm1_44925	GNALCCLQDSSSAARSK	2.368	cyclin fold protein 1 variant b	ZK353.1b
Bm1_35125	GCVNSIDQNAKARSKQI	2.304	GTP-binding regulatory protein alpha chain - starfish, putative	T07A9.7
Bm1_03585	GQIASIRRRASVPNVTN	2.205	hypothetical protein	C11H1.3 *
Bm1_15680	GSSQSYEESNRSDDLPK	1.780	hypothetical protein	M176.3 *
Bm1_01525	GQERSILSRKSQNTIEM	1.736	hypothetical protein	F54B11.5
Bm1_53985	GASLTSPLPHRPPTSVC	1.705	hypothetical protein Bm1_53985	T23F11.3b
Bm1_32000	GNGQSSNDTNIPSSHSF	1.704	hypothetical protein	F59E12.11 *
Bm1_45820	GGRCSRRAPSPTHPKLS	1.688	Protein kinase domain containing protein	ZK909.2h *
Bm1_26080	GQFLSTTASVDEESILR	1.646	Glutaredoxin family protein	ZK121.1a *
Bm1_16785	GNTGSSLTDLNLFSKGG	1.635	calcium and integrin binding family member 2	F30A10.1 *
Bm1_28345	GAYLSKPITEKISECGG	1.596	Protein phosphatase 2C containing protein	T23F11.1
Bm1_34880	GAATSGLQSQMQQEHDP	1.384	LD28933p	F20D1.1
Bm1_31655	GQQQSAFVVEIASSRFG	1.301	hypothetical protein	Y67A10A.7
Bm1_07365	GKAGSKVKNYSKIKKNN	1.146	TLD family protein	K08E7.1 *
Bm1_41730	GSCLGKKSSTTILEAVH	0.948	protein-tyrosine kinase	F49B2.5 *
Bm1_38795	GLTISGLFGRLFGKKQV	0.755	ADP-ribosylation factor 4	F57H12.1 *
Bm1_55795	GTRLSVSLEDPEVSPRT	0.189	Probable NADH-ubiquinone oxidoreductase B18 subunit	D2030.4 *
Bm1_04790	GISTSMQKDSTVLSCYG	0.183	Platelet-activating factor acetylhydrolase	C52B9.7a *
Bm1_46940	GNAAGSIKRSKSIGTWI	0.105	hypothetical protein	C52A11.2 *
Bm1_12460	GNLFGKQRPALTPVSQQ	0.076	FLJ11749-like	Y65B4A.3 *
Bm1_28970	GTTIAIKRKGTGESGKS	−0.192	G-protein alpha subunit, putative	C26C6.2 *
Bm1_51315	GICQSQEEKTMVAKSRA	−0.743	Guanine nucleotide-binding protein alpha-3 subunit, putative	E02C12.5 *
Bm1_36120	GIKSSKPKLSKEDLDFL	−0.798	Neuronal calcium sensor 2	F10G8.5 *
Bm1_17230	GNRESSTSLTSLTSLIS	−0.812	Ubiquitin carboxyl-terminal hydrolase family protein	ZK328.1b *
Bm1_54455	GKLLSKIFGKREMRILM	−1.097	ADP-ribosylation factor 6	B0336.2 *
Bm1_39660	GNMEASGREMDNPDAEE	−1.101	map kinase activated protein kinase protein 2, isoform b	F42G8.3b *
Bm1_43750	GAKISSETLIEENTYLR	−1.488	RIKEN cDNA 4933427L07	C47D12.2 *
Bm1_49765	GADGGTIPKRCELVKKK	−1.607	chromosome 20 open reading frame 43	C01A2.5 *
Bm1_50685	GALLATPACISSLACCC	−1.798	TDE2 protein	Y57E12AL.1a*
Bm1_18185	GNNQGGLHKRERALDGQ	−1.911	5′-AMP-activated protein kinase, beta subunit	Y47D3A.15

Survey of potential targets for N-terminal glycine myristoylation in *B. malayi*. Predicted myristoylated proteins in the proteome of *C. elegans* (145 clusters) were retrieved from MYRbase and used to query the *B. malayi* genome. Homologs with a BLASTP E-Value <e^−10^ were analyzed using the MYR predictor [55] to predict myristoylation sites. Protein sequences that were scored as “Reliable” with a positive score or “Twilight zone” with a negative score were retained and duplicates were discarded. *C. elegans* homologs that showed any abnormal phenotype in WormBase were marked with *. Unlikely candidates such as multi-pass integral membrane proteins were removed.

## Discussion

NMT inhibitors are being investigated by various groups for diseases including leishmaniasis [18], Chagas' disease [20]–[21], African sleeping sickness [17]–[19], [23], and malaria [17], [18], [22], [37]. The protozoan and nematode enzymes share significant similarity to human NMT yet some compounds show selectivity largely as a result of affinity and differences in the off-rates. Current drug development projects are focusing on this parameter for optimization of selectivity [23]. At the time of writing all of these programs are in the discovery phase seeking to identify clinical candidates.

To our knowledge, there have been no biochemical studies on NMT enzymes, or their substrates, from any helminth parasite. Even in the widely studied free-living nematode *C. elegans*, there is a paucity of information available. Fatty acylation of polypeptides has been experimentally demonstrated in extracts including myristoylation following metabolic labeling of *C. elegans* using [^3^H]-myristic acid [38], [39]. However, only one of many labeled proteins in the extracts was identified, namely the catalytic subunit of cAMP-dependent protein kinase [38].

In this study we established the importance of myristoylation in *C. elegans* and also in a parasitic nematode responsible for lymphatic filariasis. Our finding of highly conserved NMT sequences in the genomes of many nematode species suggests that myristoylation is likely required for the synthesis of functional nematode proteins. Comparative sequence and structural analyses between nematode and *T. brucei* NMT enzymes enabled us to consider a drug repurposing strategy using the prototypic TbNMT inhibitor, DDD85646, which has potent activity against *T. brucei* enzyme and cells in culture (nM range) [19], [23]. The compound is also active against *T. cruzi*, although ∼10–20 -fold more compound is required for enzyme inhibition, and ∼1000-fold more (µM range) to inhibit parasite growth [40]. The cause for the reduced potency against intact *T. cruzi* is not known but differences in the rate of plasma membrane turnover, cellular pharmacokinetics of drug uptake or efflux, or differences in other essential biological functions requiring N-myristoylation have been proposed [40]. DDD85646 and DDD100870 are potent inhibitors of the nematode enzymes (nM range), and possess activity against larval stages and adult worms of *B. malayi* (µM range). Interestingly, despite comparable IC_50_ values for both compounds in *C. elegans* NMT enzyme assays, only compound DDD100870 showed micromolar activity against intact *C. elegans*. The need for substantially more compound to detect activity against the nematode has also been reported in other enzyme-based screening campaigns for new filarial leads [41]–[43]. It is thought that the outermost layer of the cuticle is the main barrier to penetration of drugs, stains, and other chemicals [44], [45]. Further studies are needed to determine the physicochemical factors that favor the absorption of compounds in nematodes. This knowledge would facilitate confirming the linkage between enzyme inhibition and organism activity, and greatly expedite the discovery and optimization of anthelmintic leads.

We also pursued a genetic approach to assess the requirement of the enzyme in *C. elegans*. Knockdown of endogenous activity using RNAi resulted in severe growth defects and larval arrest, and gene deletion caused maternal effect lethality with no viable worms present in the F2 population. The importance of the gene in *B. malayi* can be inferred from our RNAi analysis since there is good concordance between the phenotypes resulting from RNAi knockdown of orthologous genes in parasitic nematodes and *C. elegans* [46]–[48].

Essential roles for NMT have also been genetically validated in other pathogens including pathogenic fungi *Candida albicans* [49] and *Cryptococcus neoformans* [50], and the kinetoplastid protozoan parasites *L. major* [51], [52], *L. donovani* [21], *T. cruzi* [40], and *T. brucei* [52]. Overexpression of NMT in *T. brucei* causes gross changes in parasite morphology, including the subcellular accumulation of lipids, leading to cell death [51]. In *Drosophila*, a null mutation of the single NMT gene causes disruption of the actin cytoskeleton and ectopic apoptosis in embryos, possibly attributed to loss of function of myristoylated tyrosine kinases [53].

Detailed studies performed in *T. brucei* indicate that inhibition of myristoylation of multiple substrates accounts for the effectiveness of NMT inhibitors in cell culture and *in vivo* [19]. While there remains incomplete knowledge of the targets of NMT in *T. brucei* and their subsequent downstream effects, trypanosomatids mainly use myristate for incorporation into glycol-phosphatidylinositol (GPI) anchors that tether the major surface glycoproteins and glycoconjugates to the external surface of the plasma membrane [54]. Recent bioinformatics analysis suggests there are in excess of 60 potential substrates in this organism [55]. Similar genome sequence analysis has identified 62 putative protein substrates for N-myristoylation in *L. major* [51], [56] and more than 40 substrates in *P. falciparum* [22]. Many of the malaria targets have been validated experimentally in a recent study [37].

In humans and other eukaryotes, including *C. elegans*, approximately 0.5–0.8% of all proteins in the genome are myristoylated [57]. The biological role of myristoylation in nematodes is poorly understood and the effect of NMT inhibition in these organisms is probably complex, likely involving the functionality of many proteins that are essential for viability. This is consistent with our genetic studies in *C. elegans* and the finding that the majority of the predicted substrates in *C. elegans* display multiple developmental defects and impaired survival in RNAi screens. We predicted potential substrates in *B. malayi* encompassing a range of functions, including *SRC-1* which encodes a non-receptor tyrosine kinase involved in cell signaling pathways specifying cell fate and division in *C. elegans* [58], [59]. The majority of the substrates are present in the proteome of one or more developmental stages of *B. malayi* [60]. Collectively, these data provide evidence for the importance of NMT activity in nematodes and provide some insight into the many diverse pathways that are likely dependent on myristoylated proteins. For further development of NMT as a drug target it would be of great interest to identify these proteins and evaluate the impact of inhibition of NMT activity resulting from genetic or chemical knockdown of the enzyme.

Myristoylation is an unexplored area for drug discovery in nematodes. Our studies have shown for the first time that NMT is a potential drug target in filarial parasites. The discovery of a related family of lead molecules, originally identified as protozoan NMT inhibitors, with activity against microfilariae and adult worms is particularly exciting and highlights the potential value of a repurposing approach to new drug discovery for filarial parasites.

## Supporting Information

Figure S1 **NMT protein sequences used to build phylogenetic tree.**
(DOCX)

Figure S2 **Alignment of the deduced amino acid sequences from various organisms.** *B. malayi* NMT and orthologs from *Loa loa* (XP_003141266.1), *O. volvulus*, *C. elegans* (NP_498326.1), *S. cerevisiae* (NP_013296.1), *L. major* (AAG38102.1), *Homo sapiens* (AAH06376.1) and *T. brucei* (EAN78792.1) were included. The alignment was generated using ClustalW and displayed with BOXSHADE. Identical (shaded black) or conserved (grey) amino acids present in at least two of the seven sequences are indicated. The amino acids that bind myristoyl-CoA (yellow) and the NMT inhibitor DDD85646 (pink) are highlighted.
(TIF)

Figure S3 **DNA sequence of native (A) and synthetic (B)** ***B. malayi*** **NMT genes.**
(DOCX)

Figure S4 **Kinetic parameters for enzyme activity.** (A) Substrate-velocity data for *C. elegans* and *B. malayi* NMT enzymes determined at varying concentrations of myristoyl CoA. Product formed (pmol per minute) is shown. (B) Lineweaver-Burk plots for CeNMT and BmNMT. The lines shown are fitted to kinetic constants determined by non-linear regression. Data from triplicate samples (Fig. S3A) were used. (C) Kinetic parameters for CeNMT and BmNMT for myristoyl CoA substrate. K_m_ is expressed in µM and the maximum velocity is expressed in number of pmol product formed per minute. K_cat_ is expressed in s^−1^ and the efficiency constant is expressed in s^−1^ µM^−1^.
(DOCX)
