# Supplementary material for: A Target Repurposing Approach Identifies N-myristoyltransferase as a New Candidate Drug Target in Filarial Nematodes
Source: PLoS Negl Trop Dis. 2014 Sep 4;8(9):e3145. doi: 10.1371/journal.pntd.0003145 (PMC4154664; doi:10.1371/journal.pntd.0003145)
Supplement: Figure S1 — NMT protein sequences used to build phylogenetic tree. (DOCX) [file pntd.0003145.s001.docx]

**Figure S1**

**NMT protein sequences used to build phylogenetic tree.**

*>B. malayi gi|170582233|ref|XP_001896037.1| N-myristoyltransferase 2 [Brugia malayi]*

MKEPPAGNIKLDDEDEKMMAAGNSKMDQNNDKENDAGDESIQNVTGRNSPTAVIDLKELTESTLMKKFEMLTVGGTSAAKHITEAQRHKYLFWDTQPVPKINEMVTENRAIEPPLDISEVREEPFSLPDPFCWCDIEINSVKELTELYTLLTENYVEDDDNMFRFDYSPEFLLWALKAPGWMKNWHCGVRAKSNGKLIAFISAIPSVIRVYDKQIKMVEINFLCVHKKLRSKRVAPVLIREITRRVNREGIFQAVFTAGVVLPKPIATCRYWHRSLNPKKLIEVKFSHLSRKMTMQRTLKLYKLPDHPRTANLVPMKKCHIDGAYGLLQCYLKKFDLSPQFTRADFEHFFMPREDVIYSYVALNEEDSKVSDLISFYSLPSSVMHHPQYKSIRAAYSFYNVATSVTLKQLINDALILARNCGFDVFNALDLMDNKEILEDLKFGIGDGNLQYYLYNWKCPDIIPEKIGLVLQ

*>W. bancrofti predicted NMT*

MKEPPAGNTKLDDENKKVMAAGDSKMDQNNDKENGDGDESTQNVAGRNSPTAVIDLKELTESALMKKFEMLTVGGTSAAKHITEAQRHKYLFWDTQPVPKINEMVTENRAIEPPLDISEVREEPFSLPDPFCWCDVEINSVKELTELYTLLTENYVEDDDNMFRFDYSPEFLLWALKAPGWMKKWHCGVRAKSNGKLIAFISAIPSVIRVYDEQIKMVEINFLCVHKKLRSKRVAPVLIREITRRVNREGIFQAVFTAGVVLPKPIATCRYWHRSLNPKKLIEVKFSHLSRKMTMQRTLKLYKLPEHPKTTNLVPMKKCHIDGAYGLLQCYLKKFNLSPQFTRADFEHFFMPREDVIYSYVALNEEDSRVSDLISFYSLPSSVMHHPQYKSIRAAYSFYNVATSVTLKQLINDALILARNCGFDVFNALDLMDNKEILEDLKFGIGDGNLQYYLYNWKCPDIVPEKIGLVLQ

*>L. loa gi|312077351|ref|XP_003141266.1| N-myristoyltransferase 2 [Loa loa]*

MEEPPIGNEKLDDENEKLMDAGDLKMAQHNGEENSDDSTQNVAGRNSPTAVIDLKELTESALMKKFEMLTVGGTSSAKHLTEAQRHKYLFWDTQPVPKINEMVTENRAIESPLDISEVREEPFSLPDPFCWSDVEIDSMKELTELYTLLTENYVEDDDNMFRFDYSPQFLLWALKAPGWMKKWHCGVRAKSSGKLIAFISAIPSVIRVYDKEVKMVEINFLCVHKKLRSKRVAPVLIREITRRVNREGIFQAAFTAGVVLPKPIATCRYWHRSLNPKKLIEVKFSHLSRKMTMQRTLKLYKLPEQPKTANLTSLKECHIDGAYGLLQCYLKKFDLSPQFTRADFEHFFMPREDVIYSYVALNEEGTKVTDLISFYSLPSSVMHHPQYKSIRAAYSFYNVATSVTLKQLINDALILARNCGFDVFNALDLMDNKEILEDLKFGIGDGNLQYYLYNWKCPDIVPEKIGLVLQ

*>O. volvulus predicted NMT*

MEELRGGKKKLDNGNEKVMAAGDSKMIQHNGEESGDDNTQNVASRNSPTAVIDLKEFTDSALMKKFEMLTVGGTSAAKHITEAQRHKYLFWDTQPVPKINEMVTENRAIEPPLDISEVREEPFSLPDPFCWYDIEINNVKELTELYTLLTENYVEDDDNMFRFDYSPQFLLWALKAPGWMKKWHCGVRAKSSGKLIAFISAIPSAIRVYDKKIRMVEINFLCVHKKLRSKRVAPVLIREITRRVNREGIFQAVFTAGVVLPKPIATCRYWHRSLNPKKLIEVKFSHLSRKMTMQRTLKLYKLPEQPKTANLTPMEKCHIDGAYSLLQCYLKKFYLAPEFTRDDFEHFFMPREDVIYSYVALNEEDNKVTDLISFYSLPSSVMHHPQYKSIRAAYSFYNVATSVTLKQLINDALILARNCGFDVFNALDLMDNKKILEDLKFGIGDGNLQYYLYNWKCPDIAPEKIGLVLQ

*>O. ochengi predicted NMT*

MEELRGGKKKLDNGNEKVMAAGDSKMIQHNGEESGDDNTQNVASRNSPTAVIDLKEFTDSALMKKFEMLTVGGTSAAKHITEAQRHKYLFWDTQPVPKINEMVTENRAIEPPLDISEVREEPFSLPDPFCWYDIEINNVKELTELYTLLTENYVEDDDNMFRFDYSPQFLLWALKAPGWMKKWHCGVRAKSSGKLIAFISAIPSAIRVYDKKIRMVEINFLCVHKKLRSKRVAPVLIREITRRVNREGIFQAVFTAGVVLPKPIATCRYWHRSLNPKKLIEVKFSHLSRKMTMQRTLKLYKLPEQPKTANLTPMEKCHIDGAYSLLQCYLKKFYLAPEFTRDDFEHFFMPREDVIYSYVALNEEDNKVTDLISFYSLPSSVMHHPQYKSIRAAYSFYNVATSVTLKQLINDALILARNCGFDVFNALDLMDNKKILEDLKFGIGDGNLQYYLYNWKCPDIAPEKIGLVLQ

*>D. immitis predicted NMT*

MDEPPARKEKLDDGKGEMMAAGDSKMIQHNGEESGDDNIQNVAGQNSPTAVIDLKEFTESTLMKKFEMLTAGGTSAAKHITEAQRHKYLFWDTQPVPKINEMVTENSAIEPPLEISEVREEPFSLPDPFCWCDIEINSAKELTELYTLLTENYVEDDDNMFRFDYSPQFLLWALKPPGWMKKWHCGVRAKSSGKLIAFISAIPSAIRVYDKKIKMVEINFLCVHKKLRSKRVAPVLIREITRRVNREGIFQAAFTAGVVLPKPIATCRYWHRSLNPKKLIEVKFSHLSRKMTMQRTLKLYKLPEQPKTANLTIMKKCHIDGAYSLLQCYLKKFYLAPEFTRDDFEHFFMPREDVIYSYVALNEEGNKVTDLISFYSLPSSVMHHPQYKSIRAAFSFYNVATSVTLKQLINDALILARNCGFDVFNALDLMDNKKILEDLKFGIGDGNLQYYLYNWKCPDILPEKIGLVLQ

*>L. sigmodontis predicted NMT*

MEEPRVGSEKLDIGNKEMVAAGDSEIAQQNGEEINDDSTQDKAGRNSPTDVIDLKEFTESALMKKFEPLTVGGTSAAKHIIEAQRHKYLFWDTQPVPKINEMVTENRAIEPPLEISEVREEPFSLPDPFCWCDVEISSVKELTELYTLLTENYVEDDDNMFRFDYSPQFLLWALKAPGWMKKWHCGVRAKASGKLIAFISAIPSVIRVYNKkIKMVEINFLCVHKKLRSKRVAPVLIREITRRVNREGIFQAAFTAGVVLPKPIATCRYWHRSLNPKKLIEVKFSHLSRKMTMQRTLKLYKLPEQPKTANLTPMRKCHIDGAYDLLQCYLKKFDLSPEFTRADFEHFFMPREDVIYSYVAVSEDDKKVTDLISFYSLPSSVMHHPQYKSIRAAYSFYNVATSVTLKQLISDALILARNCGFDVFNALDLMDNKEILEDLKFGIGDGSLQYYLYNWKCPDIAPEKIGLVLQ

*>A. viteae predicted NMT*

MEEPLAGSEKLDDENEKVMSAGDSKIMQHNGEENGDTSAQNIAGQNSPTAVIDLKELTESALMKKFEMLTVGGTSAAKHITEAQRHKYLFWDTQPVPKINEMVTENRAIEPPLDISEVREEPFSLPDPFCWCDVEINSVKELTELYTLLTENYVEDDDNMFRFDYSPQFLLWALKAPGWMKKWHCGVRAKASGKLIAFISAIPSVIRVYNKKIKMVEINFLCVHKKLRSKRVAPVLIREITRRVNREGIFQAAFTAGVVLPKPIATCRLMLLRYWHRSLNPKKLIEVKFSHLSRKMTMQRTLKLYKLPEQPKTANLTPMKKCHVGSAYSLLQCYLKKFDLSPEFTLADFEHFFMPREDVIYSYVALNEDDKKVTDLISFYSLPSSVMHHPQYKSIRAAYSFYNVATSVTLKQLINDALILARNCGFDVFNALDLMDNKEILEDLKFGIGDGNLQYYLYNWKCPDIAPEKIGLVLQ

*>A. suum gi|541042935|gb|ERG81997.1| glycylpeptide n-tetradecanoyltransferase 2 [Ascaris suum]*

MADDPADAKTPDEVSSGPETGGGEHTENPQMDGSTDVIDVKDLADPAIMKKLEMLTLGGGLATKRLSDARHHKYLFWDTQPVPKINELVTENTFIEPPLNVADVRAHPFSLPEAFHWCEVDILDEDELKELYTLLTENYVEDDDNMFRFDYSPDFLRWALQAPGWMKKWHCGVRAKSSGRLLAFISAIPSTIRVYDKVIRMVEINFLCVHKKLRSKRVAPVLIREITRRVNQEGIFQAAFTAGVILPKPIATCRYWHRSLNPKKLIEVKFSHLSRKMTMQRTLKLYKLPEAPKTPGLVALQKCDIDGAFKLLTEYLKKFALVPEFTRDDFEHFFTPKADVIYTYVVREGDGRITDLISFYSLPSSVMHHPVYKSIKAAYSFYNVATTVPLKQLINDALILAHTNGFDVFNALDLMQNKEFLEELKFGIGDGNLQYYLYNWKCPDMTPEQIGLVLQ

*>C. elegans gi|17555118|ref|NP_498326.1| Protein NMT-1, isoform a [Caenorhabditis elegans]*

MSHGHSHDGAPCGGHHGDDGAGGSRPSVNDVQALVDQLRLAGVDVSNMPNIPTAPRDMDEARSKSFQFWSTQPVPQMDETVPADVNCAIEENIALDKVRAEPFSLPAGFRWSNVDLSDEEQLNELYNLLTRNYVEDDDSMFRFDYSADFLKWALQVPGFRPEWHCGVRADSNNRLLAFIGAVPQTVRVYDKTVNMVEINFLCVHKNLRSRRVAPVLIREITRRVNVTGIFQAAFTAGIVIPKPVSVCRYYHRSLNPRKLIDVRFSHLSAKMTMARTIKLYKLPEETATRNLREMKSTDVPQVFKLLTTSLKQYSLAPVYNSEEELAHALVPKKGVVYSYVAENQNGKITDFVSFYSLPSTVMGHTTHKTIYAAYLYYYVAGSVTPKQLINDSLILANREKFDVFNALDLMHNEKIFSDLKFGKGDGNLQYYLYNWKCADMKPSQIGLVLQ

*>T. spiralis gi|339240737|ref|XP_003376294.1| glycylpeptide N-tetradecanoyltransferase [Trichinella spiralis]*

MDTIYNNHDKDRNNALPKENGDLKKSSGAKDPSPEQENVSVMTVPEYHVKQKLFTLLTLSDGYRTQPKSIEAARKKTFHFWDTQPVPKFEENVLSSEAVEPNKKIEDVRKEPYSLPEGFEWCNVDVDNEEQMEETYMLLQENYVEDEDNMFRFGYSLEFLKWALKPPGWQSDWHIGVRAKRSKKLIGFISAIPAMIRIYNRLICMVEINFLCVHKKLRSKRVAPVLIREVTRRVNLHGIFQAVYTAGVVLPRPVSTCRYWHRSLNPKKLIDVRFSQLGKNMTVQRTMKLYRLPKEVRTAGFRRITEADMEAAFLLLEEYLKKFDLAPVYSLEQFKYWFTPRPDVVDTYVVENDGVVTDFASFYTLPSSVMHHPVYKSIHAAYAFYNVATKTSLVDLMHDILVVAKNANYDVFNALDLMDNKVFLEKLKFGKGDGHLHYYLYNWRCPVIPPDKVVS

*>P. falciparum gi|6581106|gb|AAF18461.1| N-myristoyltransferase [Plasmodium falciparum]*

MNDDKKDFVGRDLYQLIRNAKDKIKIDYKFWYTQPVPKINDEFDENVNEPFISDNKVEDVRKEEYKLPSGYAWCVCDITKENDRSDIYNLLTDNYVEDDDNVFRFNYSSEFLLWALSSPNYVKNWHIGVKYESTNKLVGFISAIPIDMCVNKNIIKMAEVNFLCVHKSLRSKRLAPVLIKEITRRINLESIWQAIYTAGVYLPKPISTARYFHRSINVKKLIEIGFSCLNTRLTMSRAIKLYRIDDTLNIKNLRLMKKKDIDGLQKLLNEHLKQYNLHAIFSKEDVAHWFTPIDQVIYTYVNEENGEIKDLISFYSLPSKVLGNNKYNILNAAFSFYNITTTTTFKNLIQDAICLAKRNNFDVFNALEVMDNYSVFQDLKFGEGDGSLKYYLYNWKCASCHPSKIGIVLL

*>T. brucei gi|70833288|gb|EAN78792.1| N-myristoyl transferase, putative [Trypanosoma brucei brucei strain 927/4 GUTat10.1]*

MTDKAFTEHQFWSTQPVRQPGAPDADKVGFIMESSLDAVPAEPYSLPSTFEWWSPDVANPEDLRGVHELLRDNYVEDSESMFRFNYSEEFLRWALMPPGYHQSWHVGVRLKSNKSVLGFVAGVPITMRLGTPKMVLEKREHGEDGGEEVINDYLEPQTICEINFLCVHKKLRQRRLGPILIKEVTRRVNLMNIWHAVYTSGTLLPTPFAKGHYFHRSLNSQKLVDVKFSGIPPHYKRFQNPVAVMERLYRLPDKTKTRGLRLMEPADVPQVTQLLLKRLASFDVAPVFNEEEVAHYFLPREGVVFSYVVESPVGPGKDEENAGKASKGTPTGTKCVTGGCEKVITDFFSFYSLPSTIIGNSNHSLLKVAYVYYTAATSVSITQLVNDLLIIVKLNGFDVCNVVDIYDNGTYLKELKFSPGDGNLYYYFYNWSYPSIPANEVGLVMV

*>L. major gi|11559820|gb|AAG38102.1| N-myristoyl transferase [Leishmania major]*

MSRNPSNSDAAHAFWSTQPVPQTEDETEKIVFAGPMDEPKTVADIPEEPYPIASTFEWWTPNMEAADDIHAIYELLRDNYVEDDDSMFRFNYSEEFLQWALCPPNYIPDWHVAVRRKADKKLLAFIAGVPVTLRMGTPKYMKVKAQEKGEGEEAAKYDEPRHICEINFLCVHKQLREKRLAPILIKEATRRVNRTNVWQAVYTAGVLLPTPYASGQYFHRSLNPEKLVEIRFSGIPAQYQKFQNPMAMLKRNYQLPSAPKNSGLREMKPSDVPQVRRILMNYLDSFDVGPVFSDAEISHYLLPRDGVVFTYVVENVKKVTDFFSFYRIPSTVIGNSNYNLLNAAYVHYYAATSIPLHQLILDLLIVAHSRGFDVCNMVEILDNRSFVEQLKFGPGDGHLRYYFYNWAYPKIKPSQVALVML

*>S. cerevisiae gi|6323224|ref|NP_013296.1| Nmt1p [Saccharomyces cerevisiae S288c]*

MSEEDKAKKLENLLKLLQLNNDDTSKFTQEQKKAMKDHKFWRTQPVKDFDEKVVEEGPIDKPKTPEDISDKPLPLLSSFEWCSIDVDNKKQLEDVFVLLNENYVEDRDAGFRFNYTKEFFNWALKSPGWKKDWHIGVRVKETQKLVAFISAIPVTLGVRGKQVPSVEINFLCVHKQLRSKRLTPVLIKEITRRVNKCDIWHALYTAGIVLPAPVSTCRYTHRPLNWKKLYEVDFTGLPDGHTEEDMIAENALPAKTKTAGLRKLKKEDIDQVFELFKRYQSRFELIQIFTKEEFEHNFIGEESLPLDKQVIFSYVVEQPDGKITDFFSFYSLPFTILNNTKYKDLGIGYLYYYATDADFQFKDRFDPKATKALKTRLCELIYDACILAKNANMDVFNALTSQDNTLFLDDLKFGPGDGFLNFYLFNYRAKPITGGLNPDNSNDIKRRSNVGVVML

>*H. sapiens* gi|13623535|gb|AAH06376.1| N-myristoyltransferase 2 [Homo sapiens]

MAEDSESAASQQSLELDDQDTCGIDGDNEEETEHAKGSPGGYLGAKKKKKKQKRKKEKPNSGGTKSDSAS

DSQEIKIQQPSKNPSVPMQKLQDIQRAMELLSACQGPARNIDEAAKHRYQFWDTQPVPKLDEVITSHGAI

EPDKDNVRQEPYSLPQGFMWDTLDLSDAEVLKELYTLLNENYVEDDDNMFRFDYSPEFLLWALRPPGWLL

QWHCGVRVSSNKKLVGFISAIPANIRIYDSVKKMVEINFLCVHKKLRSKRVAPVLIREITRRVNLEGIFQ

AVYTAGVVLPKPIATCRYWHRSLNPKKLVEVKFSHLSRNMTLQRTMKLYRLPDVTKTSGLRPMEPKDIKS

VRELINTYLKQFHLAPVMDEEEVAHWFLPREHIIDTFVVESPNGKLTDFLSFYTLPSTVMHHPAHKSLKA

AYSFYNIHTETPLLDLMSDALILAKSKGFDVFNALDLMENKTFLEKLKFGIGDGNLQYYLYNWRCPGTDS

EKVGLVLQ
