# Supplementary figures and images for: A Target Repurposing Approach Identifies N-myristoyltransferase as a New Candidate Drug Target in Filarial Nematodes
Source: PLoS Negl Trop Dis. 2014 Sep 4;8(9):e3145. doi: 10.1371/journal.pntd.0003145 (PMC4154664; doi:10.1371/journal.pntd.0003145)

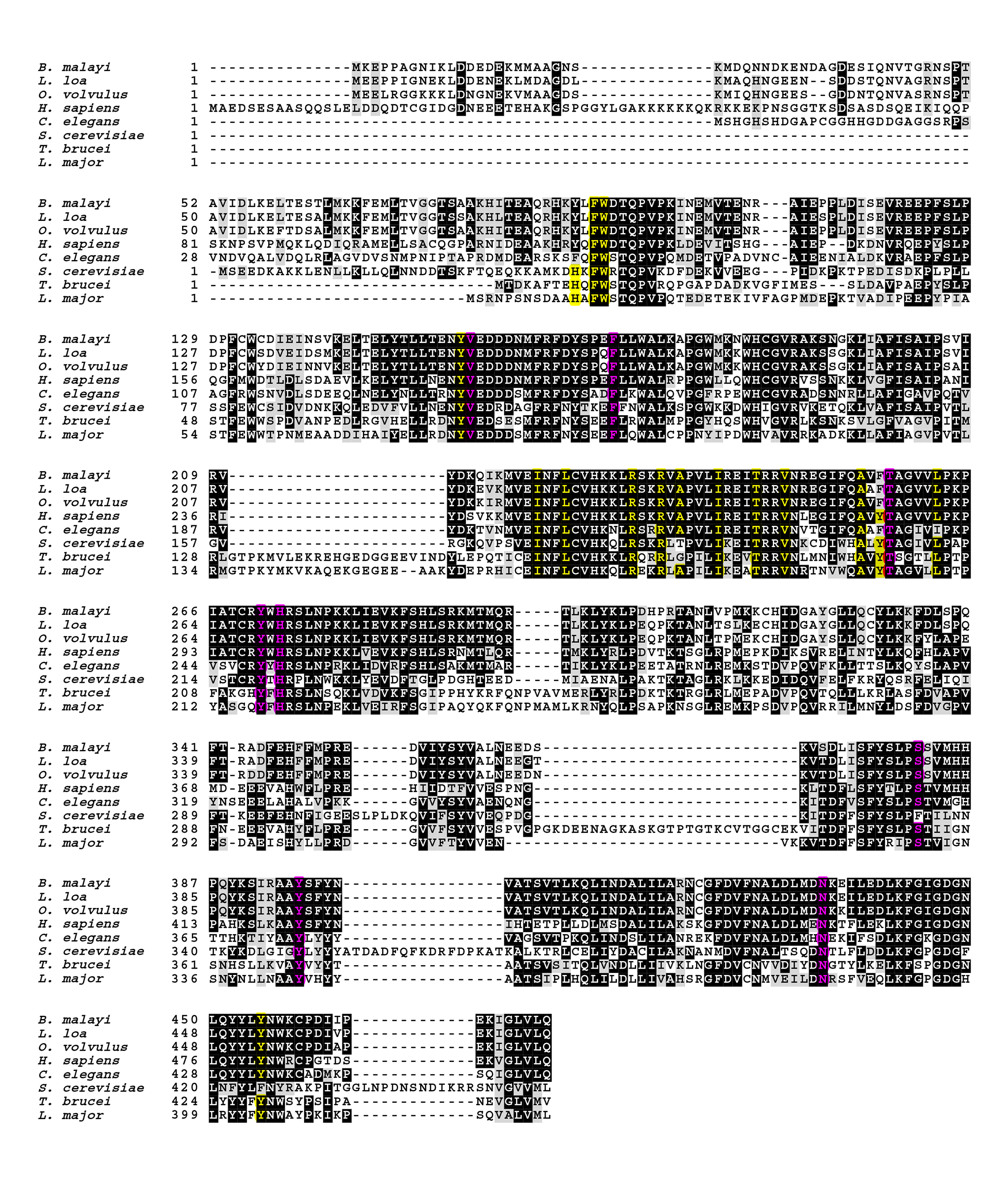

Supplement: Figure S2 — Alignment of the deduced amino acid sequences from various organisms. B. malayi NMT and orthologs from Loa loa (XP_003141266.1), O. volvulus, C. elegans (NP_498326.1), S. cerevisiae (NP_013296.1), L. major (AAG38102.1), Homo sapiens (AAH06376.1) and T. brucei (EAN78792.1) were included. The alignment was generated using ClustalW and displayed with BOXSHADE. Identical (shaded black) or conserved (grey) amino acids present in at least two of the seven sequences are indicated. The amino acids that bind myristoyl-CoA (yellow) and the NMT inhibitor DDD85646 (pink) are highlighted. (TIF) [file pntd.0003145.s002.tif]
