# Supplementary material for: A Target Repurposing Approach Identifies N-myristoyltransferase as a New Candidate Drug Target in Filarial Nematodes
Source: PLoS Negl Trop Dis. 2014 Sep 4;8(9):e3145. doi: 10.1371/journal.pntd.0003145 (PMC4154664; doi:10.1371/journal.pntd.0003145)
Supplement: Figure S3 — DNA sequence of native (A) and synthetic (B) B. malayi NMT genes. (DOCX) [file pntd.0003145.s003.docx]

**Figure S3. DNA sequence of native (A) and synthetic (B) *B. malayi* NMT genes**

**A**

ATGAAGGAACCACCAGCAGGAAACATAAAATTAGATGATGAAGATGAGAAAATGATGGCTGCTGGTAATTCAAAAATGGATCAGAATAATGATAAGGAGAATGATGCTGGTGATGAGAGCATTCAAAATGTAACTGGTCGAAATAGTCCAACTGCTGTTATCGATCTCAAAGAACTTACTGAATCTACTCTCATGAAAAAATTTGAGATGTTGACAGTTGGTGGAACATCGGCTGCTAAACATATAACCGAAGCACAACGACATAAATATCTTTTTTGGGATACGCAGCCAGTTCCAAAAATTAATGAAATGGTAACAGAGAATAGAGCTATCGAACCTCCATTGGATATATCAGAAGTACGGGAAGAGCCATTTTCATTGCCAGATCCTTTTTGTTGGTGTGATATTGAAATCAATAGTGTAAAAGAGCTTACTGAACTTTATACGCTATTGACAGAGAATTATGTGGAAGATGATGACAATATGTTTCGATTTGATTACAGTCCAGAGTTTTTGCTTTGGGCTTTAAAAGCTCCAGGATGGATGAAGAACTGGCATTGCGGTGTTCGGGCAAAATCAAATGGAAAATTGATTGCTTTTATTTCTGCTATTCCTTCAGTTATACGGGTCTATGACAAGCAAATTAAAATGGTTGAAATTAATTTTCTTTGTGTGCACAAGAAACTTCGCTCTAAAAGAGTAGCGCCAGTGTTGATTCGTGAAATCACTCGCCGAGTGAATCGAGAAGGTATCTTTCAAGCAGTATTCACAGCTGGTGTTGTTTTACCGAAACCAATTGCAACCTGCAGATACTGGCATCGATCTTTGAATCCAAAGAAACTTATTGAAGTGAAATTTTCTCATCTTTCACGGAAAATGACAATGCAGAGAACGCTGAAGCTATATAAACTTCCAGATCATCCCAGAACGGCAAACTTGGTACCGATGAAGAAATGTCATATTGATGGTGCTTATGGTTTACTACAGTGTTATCTGAAGAAGTTTGACTTATCTCCTCAATTTACTCGAGCTGATTTCGAGCATTTTTTTATGCCGCGTGAAGATGTTATCTACAGTTATGTTGCTTTGAATGAGGAAGATAGCAAAGTAAGCGATTTAATTAGCTTCTACTCATTGCCTTCATCAGTTATGCATCATCCGCAGTATAAATCAATTCGTGCAGCATATTCATTCTATAATGTCGCCACTTCAGTAACTCTTAAACAACTGATCAATGATGCTCTTATTCTTGCGCGAAATTGTGGTTTTGATGTGTTTAACGCACTTGATCTCATGGATAACAAAGAAATATTGGAAGATCTCAAGTTTGGTATTGGTGATGGAAATTTACAATATTATCTGTATAACTGGAAGTGTCCTGATATCATACCGGAGAAGATTGGTCTCGTGCTACAATAG

**B**

ATGAAAGAACCGCCGGCAGGCAATATCAAACTGGATGACGAAGACGAAAAAATGATGGCAGCAGGCAACTCTAAAATGGACCAAAATAACGATAAAGAAAATGACGCAGGTGATGAAAGTATCCAGAACGTCACCGGTCGTAATTCCCCGACGGCTGTTATTGATCTGAAAGAACTGACCGAATCAACGCTGATGAAAAAATTTGAAATGCTGACCGTTGGCGGTACCTCGGCGGCCAAACATATCACCGAAGCACAGCGCCACAAATACCTGTTTTGGGACACCCAACCGGTTCCTAAAATTAACGAAATGGTCACGGAAAATCGTGCTATCGAACCGCCGCTGGATATTAGCGAAGTTCGCGAAGAACCGTTTTCTCTGCCGGACCCGTTCTGCTGGTGTGATATTGAAATCAATAGCGTCAAAGAACTGACCGAACTGTATACCCTGCTGACGGAAAACTACGTTGAAGATGACGATAACATGTTCCGTTTCGATTACAGTCCGGAATTTCTGCTGTGGGCGCTGAAAGCCCCGGGTTGGATGAAAAATTGGCATTGCGGTGTTCGTGCGAAATCCAACGGCAAACTGATTGCATTTATCAGTGCTATTCCGTCCGTCATTCGCGTTTACGATAAACAGATCAAAATGGTTGAAATCAATTTCCTGTGTGTTCACAAAAAACTGCGTAGTAAACGCGTTGCGCCGGTCCTGATCCGTGAAATTACCCGTCGCGTTAACCGCGAAGGTATCTTTCAAGCAGTCTTCACCGCCGGCGTTGTTCTGCCGAAACCGATTGCCACGTGCCGTTATTGGCATCGCAGCCTGAATCCGAAAAAACTGATCGAAGTTAAATTTAGTCACCTGTCCCGTAAAATGACCATGCAGCGCACGCTGAAACTGTACAAACTGCCGGACCATCCGCGTACCGCAAACCTGGTTCCGATGAAAAAATGCCACATTGATGGTGCCTATGGCCTGCTGCAGTGTTACCTGAAAAAATTCGACCTGAGCCCGCAATTTACGCGTGCGGATTTCGAACATTTCTTTATGCCGCGCGAAGATGTCATCTATTCTTACGTTGCCCTGAATGAAGAAGACTCAAAAGTTTCGGATCTGATTAGCTTTTATTCTCTGCCGAGCTCTGTTATGCATCACCCGCAGTACAAATCAATTCGTGCAGCTTATTCGTTCTACAATGTTGCAACCTCTGTTACGCTGAAACAACTGATCAACGACGCACTGATTCTGGCTCGCAACTGTGGCTTTGATGTTTTCAATGCCCTGGACCTGATGGATAACAAAGAAATCCTGGAAGACCTGAAATTTGGCATTGGTGATGGCAACCTGCAGTATTACCTGTATAACTGGAAATGCCCGGACATCATCCCGGAAAAAATCGGTCTGGTTCTGCAATAA
