# Supplementary material for: A Target Repurposing Approach Identifies N-myristoyltransferase as a New Candidate Drug Target in Filarial Nematodes
Source: PLoS Negl Trop Dis. 2014 Sep 4;8(9):e3145. doi: 10.1371/journal.pntd.0003145 (PMC4154664; doi:10.1371/journal.pntd.0003145)
Supplement: Figure S4 — Kinetic parameters for enzyme activity. (A) Substrate-velocity data for C. elegans and B. malayi NMT enzymes determined at varying concentrations of myristoyl CoA. Product formed (pmol per minute) is shown. (B) Lineweaver-Burk plots for CeNMT and BmNMT. The lines shown are fitted to kinetic constants determined by non-linear regression. Data from triplicate samples (Fig. S3A) were used. (C) Kinetic parameters for CeNMT and BmNMT for myristoyl CoA substrate. Km is expressed in µM and the maximum velocity is expressed in number of pmol product formed per minute. Kcat is expressed in s−1 and the efficiency constant is expressed in s−1 µM−1. (DOCX) [file pntd.0003145.s004.docx]

**Figure S4. Kinetic parameters for enzyme activity**

**A**

|  | v(pmol product/min) | | | | | |
| --- | --- | --- | --- | --- | --- | --- |
| [s] (µM) | CeNMT | | | BmNMT | | |
| Series | Series 1 | Series 2 | Series 3 | Series 1 | Series 2 | Series 3 |
| 0.20 | 0.111 | 0.119 | 0.085 | 0.026 | 0.124 | 0.077 |
| 0.39 | - | 0.131 | 0.171 | 0.238 | 0.223 | 0.230 |
| 0.79 | 0.667 | 0.483 | 0.449 | 0.419 | 0.358 | 0.401 |
| 1.58 | 0.833 | 0.803 | 0.648 | 0.644 | 0.935 | 0.746 |
| 3.15 | 1.711 | 1.479 | 1.474 | 1.403 | 1.736 | 1.781 |
| 6.31 | 2.614 | 2.873 | 3.046 | 2.452 | 2.507 | 2.649 |
| 12.62 | 5.248 | 4.985 | 4.936 | 2.907 | 2.930 | 3.336 |
| 25.24 | 5.454 | 5.706 | 5.560 | 2.725 | 3.209 | 2.792 |

**B**

**C**

|  | CeNMT | BmNMT |
| --- | --- | --- |
| K_m_ (µM) | 12.20±0.91 | 4.38±0.12 |
| V_max_ (pmol product/minute) | 8.73±0.34 | 3.77±0.55 |
| k_cat_ (s^-1^) | 1.56 | 0.64 |
| k_cat_/K_m_ (s^-1^µM^-1^) | 0.13 | 0.15 |
